# N-Acetyl Cysteine Restores Sirtuin-6 and Decreases HMGB1 Release Following Lipopolysaccharide-Sensitized Hypoxic-Ischemic Brain Injury in Neonatal Mice

**DOI:** 10.3389/fncel.2021.743093

**Published:** 2021-11-15

**Authors:** Gagandeep Singh-Mallah, Takuya Kawamura, Maryam Ardalan, Tetyana Chumak, Pernilla Svedin, Peter G. Arthur, Christopher James, Henrik Hagberg, Mats Sandberg, Carina Mallard

**Affiliations:** ^1^Centre of Perinatal Medicine and Health, Institute of Neuroscience and Physiology, University of Gothenburg, Gothenburg, Sweden; ^2^Department of Obstetrics and Gynecology, Mie University, Tsu, Japan; ^3^School of Molecular Sciences, University of Western Australia, Perth, WA, Australia; ^4^Department of Obstetrics and Gynecology, Institute of Clinical Sciences, Sahlgrenska Academy, University of Gothenburg, Gothenburg, Sweden; ^5^Institute of Biomedicine, University of Gothenburg, Gothenburg, Sweden

**Keywords:** sirtuins, high-mobility group box 1 protein, n-acetyl cysteine, hypoxia-ischemia, lipopolysaccharide

## Abstract

Inflammation and neonatal hypoxia-ischemia (HI) are important etiological factors of perinatal brain injury. However, underlying mechanisms remain unclear. Sirtuins are a family of nicotinamide adenine dinucleotide (NAD)+-dependent histone deacetylases. Sirtuin-6 is thought to regulate inflammatory and oxidative pathways, such as the extracellular release of the alarmin high mobility group box-1 (HMGB1). The expression and role of sirtuin-6 in neonatal brain injury are unknown. In a well-established model of neonatal brain injury, which encompasses inflammation (lipopolysaccharide, LPS) and hypoxia-ischemia (LPS+HI), we investigated the protein expression of sirtuin-6 and HMGB1, as well as thiol oxidation. Furthermore, we assessed the effect of the antioxidant N-acetyl cysteine (NAC) on sirtuin-6 expression, nuclear to cytoplasmic translocation, and release of HMGB1 in the brain and blood thiol oxidation after LPS+HI. We demonstrate reduced expression of sirtuin-6 and increased release of HMGB1 in injured hippocampus after LPS+HI. NAC treatment restored sirtuin-6 protein levels, which was associated with reduced extracellular HMGB1 release and reduced thiol oxidation in the blood. The study suggests that early reduction in sirtuin-6 is associated with HMGB1 release, which may contribute to neonatal brain injury, and that antioxidant treatment is beneficial for the alleviation of these injurious mechanisms.

## Introduction

The complex etiology underlying perinatal brain injury includes, among other factors, inflammation and neonatal hypoxia-ischemia (HI) (Hagberg et al., 2015). Furthermore, inflammation, acting in combination with neonatal HI, increases the risk of perinatal brain injury, as shown in both rodents (Eklind et al., 2001, 2005; Wang et al., 2009) and larger animals (Martinello et al., 2019). Ischemic injury and neurodegeneration in adults have also been associated with oxidative stress and inflammation (Shao et al., 2020; Yang W. et al., 2020). Acetylation of histones is recognized as an important posttranslational modulation of inflammatory genes. Sirtuins are nicotinamide adenine dinucleotide (NAD)+-dependent histone deacetylases (HDACs), with several histone and non-histone targets (Houtkooper et al., 2012). Sirtuins are located in different subcellular compartments (Houtkooper et al., 2012). For instance, sirtuin-1 (SIRT1) (Li, 2014) and sirtuin-6 (SIRT6) (Zhang et al., 2017) regulate anti-inflammatory and antioxidant pathways mainly in the nucleus and cytosol, whereas sirtuin-3 (SIRT3) plays an important antioxidant role in mitochondria (Bause and Haigis, 2013). The expression and activity of SIRT1 are downregulated following HI in neonatal rats and restored by melatonin treatment (Carloni et al., 2014, 2016, 2017). In another study, melatonin-mediated protection against LPS-induced neuronal damage in the dentate gyrus of rats was shown to involve upregulation of SIRT1 and the antioxidant nuclear factor erythroid 2-related factor 2 (NRF2) pathway, which caused a reduction in inflammation and oxidative stress (Shah et al., 2017).

High mobility group box-1 (HMGB1) is an ubiquitous non-histone DNA-binding protein located in the nucleus that functions as a structural cofactor to regulate transcription (Ulloa and Messmer, 2006). Under physiological conditions, HMGB1 can act as a neurite growth factor in the immature brain (Merenmies et al., 1991; Zhang et al., 2011). However, under pathological conditions, HMGB1 is released from the nucleus into the extracellular space of activated immune cells and acts as a pro-inflammatory mediator (Wang et al., 1999b). HMBG1 translocation has also been observed in injured cells and demonstrated in animal models of adult stroke (Kim et al., 2006; Muhammad et al., 2008; Zhang et al., 2011) and neonatal HI brain injury (Zhang et al., 2016; Chen et al., 2019). HMGB1 is considered a sensitive early marker of neonatal HI brain injury, as nuclear to cytoplasmic translocation and extracellular release of HMGB1 can be detected immediately and up to 6 h after neonatal HI (Chen et al., 2019).

SIRT6 has been implicated in the prevention of the extracellular release of HMGB1. For instance, siRNA-mediated knockdown of SIRT6 was shown to increase the extracellular release of HMGB1 from SH-SY5Y neuronal cells following exposure to oxygen-glucose deprivation (Lee et al., 2013). Similar effects of SIRT6 knockdown were shown on other cell types (Kong et al., 2020). The reduced expression of SIRT6 in ischemic regions of adult rodent brain coincided with the cytosolic translocation of HMGB1 (Lee et al., 2013).

The effect of neonatal brain injury on SIRT6, in association with HMGB1 translocation, is not known. Therefore, the aim of this study was to investigate SIRT6 expression and the extracellular release of HMGB1 in a neonatal model of LPS-sensitized HI brain injury. Furthermore, the effect of neuroprotection on the expression of SIRT6 and HMGB1 was assessed using N-acetyl cysteine (NAC), as it is a well-known antioxidant and has previously been demonstrated to improve cellular redox status (Adams et al., 2021) and reduce brain injury in a neonatal animal model that combined LPS and hypoxia-ischemia (Wang et al., 2007).

## Experimental Procedures

### Animals

C57BL/6J mice (6− to 8-week-old), bought from Janvier Laboratories (Le Genest-Saint-Isle, France) and Charles River Laboratories (Sulzfeld, Germany), were bred at the Laboratory for Experimental Biomedicine (Sahlgrenska Academy, University of Gothenburg, Sweden). The animals were kept in a 12 h light-dark cycle with *ad libitum* access to standard laboratory chow (B&K, Solna, Sweden) and water. All the animal experiments were approved by the Gothenburg Animal Ethics Committee. In all the experiments, animals from at least three litters were used, and pups within each litter were equally distributed among the treatment groups, including distribution of sex of pups whenever possible.

### Hypoxia-Ischemia, Lipopolysaccharide, and N-Acetyl Cysteine Treatment

The day of birth was considered post-natal day (PND) 0. On PND 8, mice were randomly selected for injection with either lipopolysaccharide (LPS) (O55:B5; *i.p.*; 0.3 mg/kg in endotoxin-free saline) or an equivalent volume of endotoxin-free saline (SAL). In a separate cohort, PND 8 pups were administered LPS or SAL as above and 14 h after injection (i.e., on PND 9) the pups were also exposed to hypoxia-ischemia (HI) using the modified Rice-Vanucci model as described before (Wang et al., 2009). Briefly, the left common carotid artery was ligated, and the mice were exposed to 10% O_2_ for 30 min in a humidified incubator maintained between 35.5 and 36.5°C.

N-acetyl cysteine or vehicle (VEH, i.e., endotoxin-free saline) was administered at the time of LPS injection and immediately before HI in animals subjected to the combination of LPS and HI. The dose of NAC (200 mg/kg in endotoxin-free saline; *i.p.*; Sigma-Aldrich, St. Louis, MO, United States) was selected based on our previous study on neonatal rats (Wang et al., 2007).

### Tissue Collection for Biochemical Analysis

Mice were deeply anesthetized *via* intraperitoneal administration of pentobarbital (Pentacour) and intracardially perfused with 0.9% saline. Brain tissue was collected on a cold plate and frozen in dry ice followed by storage at −80°C. The tissue samples were homogenized using a lysis buffer containing Tris-HCl (25 mM, pH 7.9), NaCl (100 mM), EDTA (5 mM), NP40 (1%), sodium butyrate (0.1 mM), Nam (5 mM), protease inhibitor (1%), phosphatase inhibitor, and PBS (pH 7.4). The lysate was analyzed by Western blotting and quantitative real-time PCR. The fraction for Western blotting was centrifuged at 15,000 *g* for 5 min at 4°C, and the supernatant was stored at −80°C. The remaining fractions were directly stored at −80°C. Protein concentration in the lysates was measured using a bicinchoninic acid protein assay kit (Thermo Fisher Scientific, Waltham, MA, United States).

### Quantitative Real-Time PCR

Total RNA was extracted from the brain samples using the RNeasy mini kit (Qiagen, Solna, Sweden) and measured in a spectrophotometer at 260-nm absorbance (NanoDrop 2000/2000c; Thermo Fisher Scientific, Rockford, IL, United States). Total RNA (500 ng) from each sample was used for first-strand cDNA synthesis according to the instructions of the manufacturer (QuantiTect reverse transcription kit; Qiagen, Solna, Sweden). In order to determine mRNA expression, the cDNA samples were further processed by quantitative real-time (qRT)-PCR. Each PCR reaction (20 μl) contained 10 ng of cDNA in a 5-μl volume, 10 μl of QuantiFast SYBR Green PCR master mix (Qiagen, Solna, Sweden), 2 μl of PCR primers, and 3 μl of H2O, to make a final reaction volume of 20 μl. The PCR primer was Mm_Sirt6_1_SG QuantiTect Primer Assay QT00112700 (Qiagen, Solna, Sweden). The amplification protocol comprised an initial 5-min denaturation at 95°C, followed by 40 cycles of denaturation for 10 s at 95°C and annealing/extension for 30 s at 60°C on LightCycler 480 (Roche, Stockholm, Sweden). Melting-curve analysis was performed to ensure that only one PCR product was obtained. For quantification and estimation of amplification efficiency, a standard curve was created using increasing concentrations of cDNA. The amplification transcripts were quantified with the relative standard curve and normalized to the concentration of cDNA in each RT sample measured using the Quant-iTTM 338 OliGreen ssDNA Assay Kit 339 (Invitrogen, Waltham, MA, United States) in accordance with (26). Data were normalized to geomean of Hprt1 and Ywhaz genes.

### Western Immunoblotting

Tissue lysates were mixed with 4× Laemmli Sample buffer (Bio-Rad Laboratories, Hercules, CA, United States) and 10% *β*-Mercaptoethanol (Merck, Darmstadt, Germany), and diluted using PBS to obtain same protein concentration across all samples. The samples were heated at 95°C for 5 min before 10–20 μg of each was loaded on a 4–20% reducing gel (Criterion^*TM*^ TGX Stain-Free^*TM*^ Precast Gels; Bio-Rad Laboratories, Hercules, CA, United States), and transferred to a 0.2-μm nitrocellulose membrane (Bio-Rad Laboratories, Hercules, CA, United States). Stain-free blots were imaged using a Gel Doc XR Plus system (Bio-Rad Laboratories, Hercules, CA, United States), and images were saved for normalization of immunoreactive bands to the total protein. Membranes were blocked with TBS-Tween (TBS-T) buffer (30 mM/L Tris-HCl, 100 mM/L NaCl, and 0.1% Tween: pH 7.45) containing 5% nonfat milk for 1 h at room temperature. After washing with TBS-T, the membranes were incubated overnight at 4°C with the following primary antibodies: sirt1 mouse monoclonal antibody (1:500, CST 8469; Cell Signaling, Leiden, WZ, Netherlands) and Sirt6 rabbit monoclonal antibody (1:500, CST 12486; Cell Signaling, Leiden, WZ, Netherlands). The membranes were washed with TBS-T and incubated with appropriate peroxidase-labelled secondary antibody (1:5,000; Vector Laboratories, Burlingame, CA, United States) in 5% nonfat milk for 1 h. Immunoreactive bands were visualized using an ECL substrate (Bio-Rad Laboratories, Hercules CA, United States) and a Gel Doc XR Plus system (Bio-Rad Laboratories, Hercules, CA, United States), and quantified using the Image Lab^*TM*^ software after normalizing to total protein (Bio-Rad Laboratories, Hercules CA, United States).

### Multiplex Protein Analysis

Cytokines and chemokines in lysates from hippocampus were detected with a cytometric bead array using a Bio-Plex Pro mouse cytokine 9-plex assay (Bio-Rad Laboratories, Hercules CA, United States) and analyzed with a Luminex 200 system (Bio-Rad Laboratories, Hercules CA, United States) according to the instructions of the manufacturer and as previously described (Lai et al., 2020).

### Thiol Oxidation

The oxidation of plasma albumin was measured as previously described (Lim et al., 2020) with the following modifications: blood samples were collected by heart puncture and applied onto a PerkinElmer 226 Spot Saver RUO Card containing polyethylene glycol maleimide. Cards were stored with silica gel desiccant for transport to the University of Western Australia. Albumin was extracted into 0.05% Tween 20 in 20 mM phosphate with further binding to Cibacron Blue F3GA agarose. Albumin was eluted with 25 μl of 1.4 M NaCl in 20 mM phosphate buffer, pH 7.4. Gel electrophoresis, imaging, and calculation of total albumin oxidation were performed as previously described (Lim et al., 2020).

### Tissue Processing for Immunohistochemical Staining

The mice were deeply anaesthetized *via* intraperitoneal administration of pentobarbital (Pentacour) (60 mg/ml). The brains were removed from the skull and immersed in 6% buffered formaldehyde (Histofix; Histolab products AB; Västra Frölunda, Sweden) and stored at 4°C for 1 week. At the time of tissue processing, the brains were placed in a cryoprotective solution containing 30% (w.v1) sucrose for 48 h followed by placement on copper blocks for freezing in cold isopentane (Sigma Aldrich, Darmstadt, Germany). Free-floating 60-μm thick sections were cut coronally on a cryostat (Leica CM 3050 S; Leica, Wetzlar, Germany) and selected based on a systematic sampling principle and a section-sampling fraction of 1/6 (Gundersen, 2002) by selecting the first section of each series randomly using a random table.

### Immunohistochemistry

The free-floating 60-μm thick sections were washed in phosphate-buffered saline (PBS, 0.1 M, pH 7.2, 30 min) followed by blocking with endogenous peroxidase (3% H_2_O_2_ in PBS, 30 min). Antigen retrieval was performed using 0.01 M citrate buffer (40 min at 85°C). Thereafter, the sections were rinsed in PBS (2 min × 15 min) and placed in 4% goat serum in PBS for 1 h. In the next step, the sections were incubated with a monoclonal rabbit anti-HMGB1 and anti-sirt6 (1:350, Ref# ab79823; 1:500, D8D12 CST; Abcam, Cambridge, United Kingdom) overnight at 4°C, followed by washing in PBS, incubation in polyclonal secondary goat-anti-rabbit biotinylated (1:200;Vector Laboratories, Olean, NY, United States) for 2 h, and washing in PBS (2 min × 15 min) and ABC elite (avidin-biotin-complex; Vector Laboratory, Burlingame, CA, United States) for 1 h at room temperature. Immunolabeling was performed using a 3,3-diaminobenzidine solution (Acros Organics, Geel, Belgium). Finally, the sections were mounted on gelatine-coated slides, dried, re-hydrated in demineralized water, dehydrated through a graded series of alcohol (95% and 99%), cleared in xylene, and coverslipped.

### Measurement of Hippocampal High Mobility Group Box-1 Positive Area

Quantification of the area in hippocampus that exhibited HMGB1 translocation and release was performed on the HMGB1-stained sections by point counting using the Cavalieri estimator (Anzabi et al., 2018) and using a 10× objective lens under light microscope modified for stereology with a digital camera (Leica DFC 295; Leica, Wetzlar, Germany) and the newCAST^*TM*^ software (Visopharm, Hørsholm, Denmark) (Figure 5). The following formula was used to calculate the volume of the HMGB1-positive area:

**FIGURE 1:**
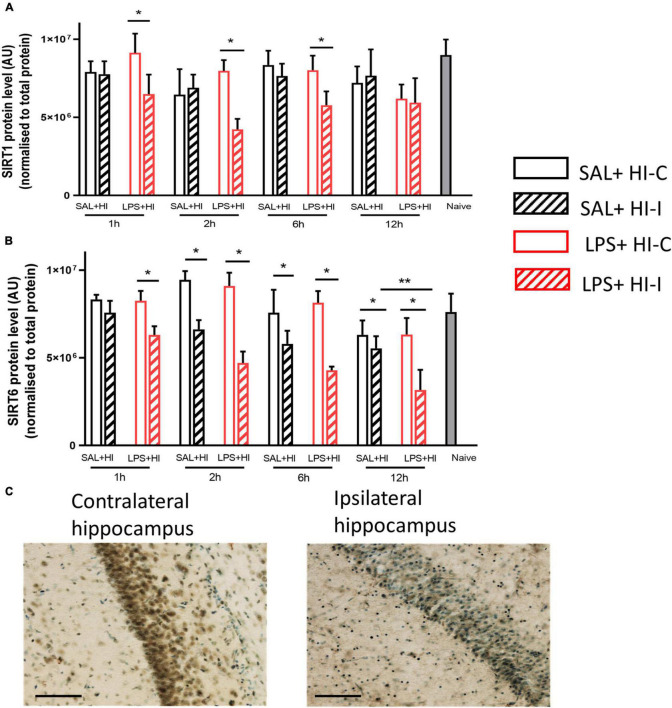
Sirtuin 1 and sirtuin 6 protein expression after saline (SAL) + hypoxia-ischemia (HI) and lipopolysaccharide (LPS) + HI. Western blots of **(A)** sirtuin-1 **(B)** and sirtuin-6 were performed 1–12 h after SAL + HI or LPS+HI in injured (ipsilateral) and uninjured (contralateral) hippocampi. *N* = 5–6/group, *p < 0.05, **p < 0.01. Paired Wilcoxon signed-rank test (paired data) and Mann–Whitney *U* (non-paired data) were applied. Example of sirtuin 6 staining in the CA1 region of hippocampus (ipsi- and contralateral) 6 h after LPS + HI (20× objective lens), scale bar = 100 μm **(C)**.

**FIGURE 2:**
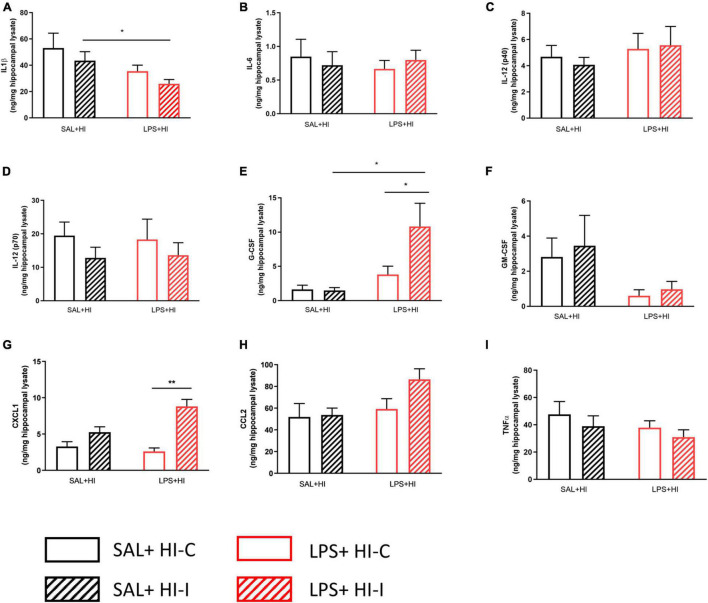
Cytokine and chemokine levels following SAL + HI and LPS + HI. Multi-bead array analysis (9-plex) was applied to hippocampal lysate from ipsi- and contralateral hemispheres 2h after SAL + HI or LPS + HI. Protein expression for **(A)** IL-1β, **(B)** IL-6, **(C)** IL-12 (p40), **(D)** IL-12 (p70), **(E)** G-CSF, **(F)** GM-CSF, **(G)** CXCL1, **(H)** CCL2, and **(I)** TNF-α **(I)** was analyzed. *n* = 5–6/group, *p < 0.05; **p < 0.01. Paired *t*-test (paired data) and independent *t*-test (non-paired data) were performed.

**FIGURE 3:**
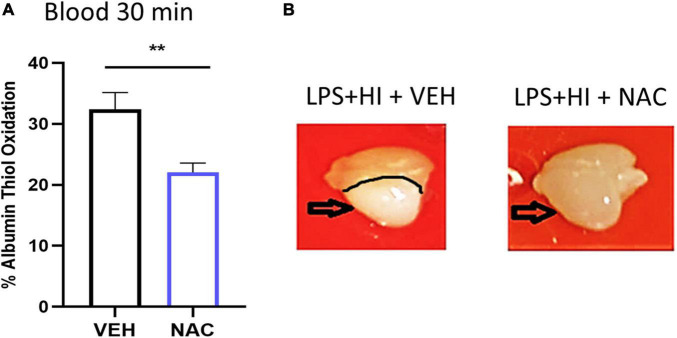
Effect of N-acetyl cysteine (NAC) treatment on thiol oxidation and brain injury after LPS + HI. Animals were subjected to LPS + HI and administered VEH (saline) or NAC treatment. **(A)** Thiol oxidation was analyzed 30 min after LPS + HI in blood. *n* = 7/group, **p < 0.01. Independent *t*-test was applied. Brains were removed and photographed 12 h after LPS + HI. **(B)** Typical example of brain injury appearance in VEH- and NAC-treated animals.

**FIGURE 4:**
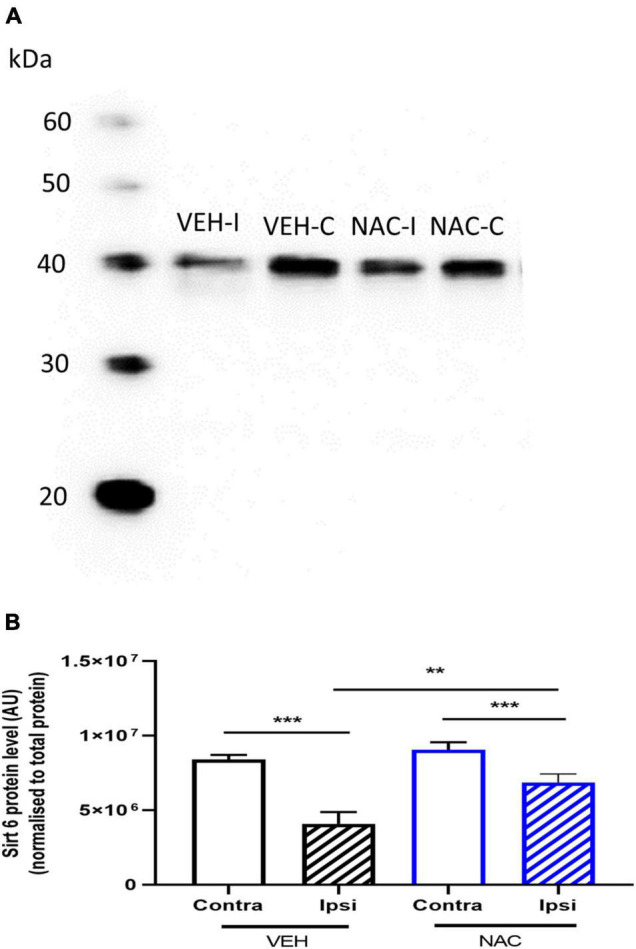
Sirtuin 6 protein expression in the hippocampus following LPS + HI with or without NAC treatment. **(A,B)** Animals were subjected to LPS + HI, and sirtuin 6 protein expression was analyzed in ipsilateral (ipsi) and contralateral (contra) hippocampi by Western blot 12 h after LPS + HI with NAC or VEH treatment. *n* = 10–12/group **p < 0.01, ***p < 0.001, *n* = 10–12/group. Paired *t*-test (paired data) and independent *t*-test (non-paired data) were performed.

**FIGURE 5:**
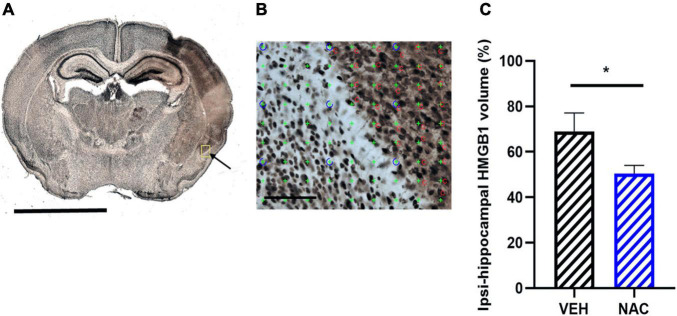
Immunohistochemistry of high mobility group box-1 (HGMB1) release after LPS + HI with or without NAC treatment. **(A)** Representative image (5× objective lens) of neonatal mouse brain section stained with HMGB1 at the hippocampal level 6 h after LPS + HI and the area of HMGB1 translocation and release in the ipsilateral brain hemisphere (arrow), scale bar = 2.5 mm. **(B)** Application of point counting on 60-μm thick coronal HMGB1-stained sections for measuring the area with extracellular HMGB1 release, scale bar = 100 μm. **(C)** From area measurements, the percent of HMGB1 extracellular volume was calculated on hippocampal ipsilateral side and contralateral side in the VEH and NAC groups. *n* = 6–8, *p < 0.05. Independent *t*-test was performed.


$$V=\Sigma \,P\cdot \left(\frac{a}{p}\right)\cdot T\cdot \frac{1}{S\,S\,F}$$

where ΣP is the total number of the points hitting the region of interest per animal, (a/p) is the area per test point; T is the section thickness (60 μm), and SSF is the section sampling fraction (1/6). The size of the HMGB1-positive area was presented as % of the size of the hippocampus.

### Statistical Analysis

Data are presented as mean ± SD. All the statistical analyses were conducted using the SPSS statistical software (SPSS Statistics 21; IBM, Armonk, NY, United States). Graphs were created using Prism 8 (GraphPad Software Inc., San Diego, CA, United States). Prior to statistical tests, normal distribution of data was checked by making a Q–Q plot of the data. The variance homogeneity of data was also examined by Levene’s test. Data with normal distribution were compared between two independent groups (Saline/HI and LPS/HI) by independent *t*-test, and those with non-normal distribution were analyzed by Mann–Whitney U test. For analysis between ipsilateral and contralateral sides, the data were considered as paired. In such analysis, normally distributed data were analyzed by paired *t*-test, and non-normally distributed data were analyzed by paired Wilcoxon signed-rank test. *P* < 0.05 was considered statistically significant.

## Results

### Lipopolysaccharide Alone Does Not Affect Sirtuin-6 mRNA or Protein Expression

In order to understand if LPS by itself affects sirtuin-6 expression, we measured the *sirt6* mRNA and SIRT6 protein expression in the brain 12 h after LPS injection. There was no difference in mRNA expression (Supplementary Figure 1A, *p* > 0.05) or in protein expression (Supplementary Figure 1B, *p* > 0.05) in the brain after LPS.

### Reduced Sirtuin 1 and Sirtuin 6 Protein Expression Following Lipopolysaccharide + Hypoxia-Ischemia

To analyze temporal changes in the expression of SIRT1 and SIRT6 proteins in response to an injurious event, we collected hippocampal tissues at 1, 2, 6, and 12 h after SAL + HI and LPS + HI (*n* = 5–6 per group and time point). The hippocampus was analyzed, as it is the most sensitive brain region in infection-induced sensitization of HI injury in neonatal mice (Lai et al., 2020). SIRT1 protein levels significantly decreased in the ipsilateral hippocampus compared with the contralateral side at 1, 2, and 6 h in the LPS + HI group (Figure 1A). There were no significant changes in SIRT1 levels between hemispheres following SAL + HI (Figure 1A). Analysis of SIRT1 protein levels in the ipsilateral hippocampus in SAL + HI and LPS + HI, within each time-point, showed no difference between the two groups (Figure 1A).

Sirtuin 6 protein expression was significantly decreased in the ipsilateral hippocampus compared with the contralateral side at all time points (1–12 h) after LPS + HI, while SIRT6 protein expression in the hippocampus was reduced at 2–12 h after SAL + HI (Figure 1B). Analysis of SIRT6 protein levels between the ipsilateral hippocampus in SAL + HI and LPS + HI within each time point showed significantly lower level of SIRT6 protein at 12 h after LPS + HI, while no changes at other time points were observed (Figure 1B).

Immunohistochemical staining for SIRT6 was evident in neurons in the contralateral CA1 region of the hippocampus, while staining was markedly reduced in the ipsilateral hippocampus 6 h after LPS + HI (Figure 1C).

### Cytokine and Chemokine Response in Hippocampus Following Saline + Hypoxia-Ischemia and Lipopolysaccharide + Hypoxia-Ischemia

As both SIRT1 and SIRT6 protein expressions changed already 1–2 h after LPS + HI, we next used a cytometric bead array to measure a panel of cytokine and chemokine proteins in the hippocampus 2 h following SAL + HI and LPS + HI. Cytokines IL-1β, IL-6, IL-12 (p40), IL-12 (p70), GM-CSF, TNF-*α*, and the chemokine CCL2 were unchanged in the ipsilateral compared with contralateral hippocampi in the SAL + HI and LPS + HI groups (Figure 2, *n* = 5–6/group, *p* > 0.05). In contrast, G-CSF and CXCL1 were increased in the ipsilateral hippocampi compared with the contralateral hippocampi following LPS + HI (Figure 2, *n* = 5–6/group, *p* = 0.026 and *p* = 0.002, respectively). Analysis of cytokine and chemokine proteins in the ipsilateral hippocampus following SAL + HI and LPS + HI showed significant difference in IL-1β, G-CSF, and CXCL1 between the two groups (Figure 2).

### N-Acetyl Cysteine Treatment Reduces Thiol Oxidation and Brain Injury After Lipopolysaccharide + Hypoxia-Ischemia

To investigate the effects of an antioxidant/anti-inflammatory compound on SIRT6 and HMGB1 in our model, we used NAC, as we have previously shown this to be protective in neonatal rats following inflammation-induced HI injury (Wang et al., 2007; Mottahedin et al., 2020). First, we confirmed the antioxidant effect of NAC and show reduced thiol oxidation in blood 30 min following NAC compared with VEH-treated LPS + HI animals (Figure 3A, *n* = 7/group, *p = 0*.007). Inspection of brain tissue 12 h after LPS + HI in VEH- and NAC-treated animals showed clear reduction in tissue pallor (indicative of reduced injury) in the NAC-treated animals (Figure 3B).

### N-Acetyl Cysteine Treatment Restores Sirtuin 6 Protein Levels

To determine the effects of NAC on the expression of SIRT6 protein, hippocampi were collected from NAC- and VEH-treated LPS + HI mice 12 h after HI. SIRT6 levels were reduced in the ipsilateral hippocampus compared with the contralateral side in both VEH- and NAC-treated animals (Figure 4, *n* = 10–12/group, *p* = 0.001, *p* = 0.001, respectively). The reduction in SIRT6 in the ipsilateral hippocampus was significantly less in the NAC-treated animals compared with the SAL-treated mice (Figure 4, *n* = 10–12, *p* = 0.009).

### N-Acetyl Cysteine Treatment Reduced Hippocampal High Mobility Group Box-1 Translocation and Release After Lipopolysaccharide + Hypoxia-Ischemia

Sirtuin 6 has been suggested as a regulator of HMGB1 release. Thus, we investigated the immunohistochemical expression of extracellular HMGB1 in the hippocampus after LPS-HI with or without NAC treatment. Extracellular HMGB1 positive areas, as determined by unbiased stereological method, were apparent in the ipsilateral hemisphere in both groups (Figures 5A,B). We found that the area of extracellular HMGB1 staining in the ipsilateral hippocampus was significantly smaller in the NAC group compared with the VEH group (Figure 5C, *n* = 6–8/group, *p* = 0.041).

## Discussion

Sirtuin 6 has been relatively little studied compared with other members of the sirtuin family (Klein and Denu, 2020). However, SIRT6 has been implicated as a therapeutic target in diverse conditions such as cardiovascular (Saiyang et al., 2021), Alzheimer’s (Mohamad Nasir et al., 2018), cancer (Fiorentino et al., 2021), and several other age-related diseases (Zhao et al., 2020). As perinatal brain injury and underlying mechanisms significantly differ from the adult (Mallard and Vexler, 2015), this study has focused on injury mechanisms in the neonatal brain. This is the first study to have investigated SIRT6 in a neonatal model of inflammation-induced brain injury. We demonstrate that the expression of SIRT6 protein is reduced and that the release of extracellular HMGB1 is increased in the injured ipsilateral hemisphere after LPS + HI. Treatment with the antioxidant NAC normalized these changes, which were accompanied by amelioration of brain injury. These results suggest that SIRT6 has a protective effect on the developing brain, effects at least partially linked to the regulation of HMGB1 extracellular release.

Sirtuin 6 deficiency in nonhuman primates results in developmental delay, including delay in neuronal differentiation (Zhang et al., 2018). It has been suggested that SIRT6 is a regulator of dendrite morphogenesis in rat hippocampal neurons and, therefore, important in development (Matsuno et al., 2021). SIRT6 has also been shown to protect the adult brain from cerebral ischemia/reperfusion injury (Zhang et al., 2017). While in this study LPS by itself did not alter *sirtuin-6* mRNA or SIRT6 protein levels in the neonatal brain, SIRT6 expression in the injured hemisphere was reduced already 1 h after LPS + HI, and this reduction became more prominent with time. A similar response in SIRT6 was observed after SAL + HI, but started later (2 h) and with less marked changes. We know from our previous studies that brain injury is significantly more pronounced following LPS + HI compared with SAL+HI (Wang et al., 2009), which has also been shown in piglets (Martinello et al., 2019). Thus, the data suggest that the magnitude of reduction in SIRT6 is associated with the degree of injury. Similarly, we found significantly reduced expression of the SIRT1 protein, however, only in association with more severe LPS + HI-induced injury but not after SAL + HI. In support, the early regulation of SIRT1 has been shown previously after prolonged severe neonatal HI (2.5 h) (Carloni et al., 2017).

We observed increased HMGB1 extracellular staining in brain regions known to be injured following neonatal LPS + HI (Wang et al., 2009). Clinical studies have demonstrated that HMGB1 levels are elevated in the blood of neonates with perinatal asphyxia (Okazaki et al., 2008) and in the umbilical blood of neonates suffering from hypoxic-ischemic encephalopathy (Nakamura et al., 2013). HMGB1 extracellular release in the brain has been observed after cerebral ischemia in fetal sheep (Zhang et al., 2016) and in neonatal rat brain after cerebral hypoxia-ischemia (Chen et al., 2019). HMGB1 can be passively released during various forms of cell death, such as pyroptosis, apoptosis, autophagy, necroptosis, and necrosis (Scaffidi et al., 2002). Increased extracellular HMGB1 was recently linked to HI-induced brain damage in the hippocampus of neonatal mice, and it was shown that treatment with an HMGB1 inhibitor reversed the HI-induced loss of gray and white matter in the hippocampus and reduced neurobehavioral impairments (Le et al., 2020).

Increased extracellular HGMB1 staining was observed in the same brain regions where we found decreased SIRT6 expression, suggesting a possible mechanistic link. Similarly, decreased expression of SIRT6 was associated with the release of HGMB1 after adult cerebral ischemia (Lee et al., 2013). In that study, Lee et al. showed that *Sirtuin6* gene knockdown by siRNA increased HMGB1 translocation. While the mechanisms were not fully elucidated, co-immunoprecipitation experiments suggested that SIRT6 does not interact directly with HMGB1 (Lee et al., 2013). An alternative mechanism is that decreased SIRT6 leads to elevated chromatin acetylation, which in turn can trigger HMGB1 release and inflammation (Scaffidi et al., 2002). Another possibility is that SIRT6 affects the nuclear retention of HMGB1 through the regulation of NF-kB activity. Under normal conditions, SIRT6 can maintain inflammatory homeostasis through interaction with the NF-kB subunit RelA, leading to deacetylation of histone H3K9 on NF-kB target gene promoters, thereby decreasing transcription through NF-kB (Kawahara et al., 2009; Ha et al., 2011). However, in this study, decreased SIRT6 expression was not reflected in, at least, early expression of cytokines and chemokines, except for an increase in G-CSF and CXCL1 in the injured ipsilateral hemisphere. Potentially, SIRT6 levels may be better related to the cytokine response at later time points after HMGB1 release. However, another alternative explanation is that a lowered level of SIRT6 results in decreased ERK and IGF-1 signaling known to be protective in HI-induced neonatal brain damage (Gluckman et al., 1992; Brywe et al., 2005). ERK can regulate the translocation of HMGB1, and SIRT6-deprived cells have reduced ERK (Matsuno et al., 2021); the effect of ERK phosphorylation on HMGB1 release does not affect the production of cytokines (Zhou et al., 2013). With respect to the brain, the ERK inhibitor U0126 blocked HMGB1 release from astrocytes (Hayakawa et al., 2010). Obviously, the relationship between SIRT6 and HMGB1 release is complex and can be multifactorial; however, it is possible that decreased SIRT6, at least partly, causes increased release of HMGB1 *via* lack of ERK phosphorylation.

High mobility group box-1 has been shown to act as a mediator of endotoxemia in mice and is regarded as a damage-associated molecular pattern (DAMP) molecule, causing inflammatory responses in various diseases (Wang et al., 1999a; Lotze and Tracey, 2005; Andersson and Tracey, 2011). Once in the extracellular space, HMGB1 can activate an array of innate immune receptors, such as RAGE and toll-like receptors (TLR) 2 and 4 (Yang H. et al., 2020). The mRNA and protein expression of RAGE is increased after neonatal hypoxia-ischemia (Pichiule et al., 2007), and we have shown increased expression of TLR2 (Stridh et al., 2011). The effects on these receptors are dependent on the oxidation status of HMGB1 where the mixed form with one disulfide bridge and one reduced –SH group is the variant that activates TLRs (Kwak et al., 2020). Thus, while the mechanism of translocation from the nucleus and release to the extracellular space under ischemic conditions is not fully clear, it may include the oxidation of HMGB1 (Kwak et al., 2020). The importance of partial oxidation of HMGB1 as a trigger of nuclear to cytoplasmic translocation points toward a site of action of antioxidants, although this is not well explored. The levels of the SIRT6 protein have been shown to be reduced by H_2_O_2_-induced oxidative stress (Liu et al., 2014). It follows that part of the antioxidant protective effects of NAC against LPS+HI injury could be exerted *via* blockage of a decrease in SIRT6 that has been shown to preserve nuclear HMGB1 as well as the maintenance of the reduced form of HMGB1. Both of these effects would keep HMGB1 in the nucleus. Our data demonstrating that NAC treatment normalized SIRT6 and reduced extracellular HMGB1 release support such a notion.

We acknowledge several limitations of the study, such as the study groups were not dimensioned to detect sex differences and there was no long-term follow-up on sirtuin and HMGB1 regulation. Furthermore, NAC treatment was only administered prior to HI; thus, the effects of NAC on sirtuins and HMGB1 solely post-HI could not be assessed.

In conclusion, LPS-sensitized HI caused decreased SIRT6 levels and increased release of HMGB1 in the neonatal brain. Treatment with NAC was protective and restored SIRT6 and blocked the release of HMGB1. Further studies are needed to clarify the detailed relationship between SIRT6 and neonatal brain injury, but our data indicate that SIRT6 could be a target for therapeutic intervention against LPS-sensitized HI-induced brain damage by reducing HMGB1 release.

## Data Availability Statement

The original contributions presented in the study are included in the article/Supplementary Material, further inquiries can be directed to the corresponding author/s.

## Ethics Statement

The animal study was reviewed and approved by Gothenburg Animal Ethics Committee (No. 663/2017).

## Author Contributions

CM, MS, HH, GS-M, and TK designed the study. MA, GS-M, TK, PS, TC, PA, and CJ performed the experiments, data collection, and data analysis. CM, MS, GS-M, and TK interpreted the results and wrote the manuscript. All authors provided the conceptual advice, commented on the manuscript, and approved the final version of the manuscript for submission.

## Conflict of Interest

GS-M is currently an employee of AstraZeneca. The remaining authors declare that the research was conducted in the absence of any commercial or financial relationships that could be construed as a potential conflict of interest.

## Publisher’s Note

All claims expressed in this article are solely those of the authors and do not necessarily represent those of their affiliated organizations, or those of the publisher, the editors and the reviewers. Any product that may be evaluated in this article, or claim that may be made by its manufacturer, is not guaranteed or endorsed by the publisher.

## Supplementary Material

Supplementary Figure 1 Sirtuin 6 expression in the brain after lipopolysaccharide (LPS) treatment. Sirtuin 6 mRNA expression in the brain was assessed by **(A)** qPCR and protein expression by **(B)** Western blot 12 h after LPS. *n* = 10–17/group. Independent *t*-test was performed.
