# Supplementary figures and images for: N-Acetyl Cysteine Restores Sirtuin-6 and Decreases HMGB1 Release Following Lipopolysaccharide-Sensitized Hypoxic-Ischemic Brain Injury in Neonatal Mice
Source: Front Cell Neurosci. 2021 Nov 15;15:743093. doi: 10.3389/fncel.2021.743093 (PMC8634142; doi:10.3389/fncel.2021.743093)

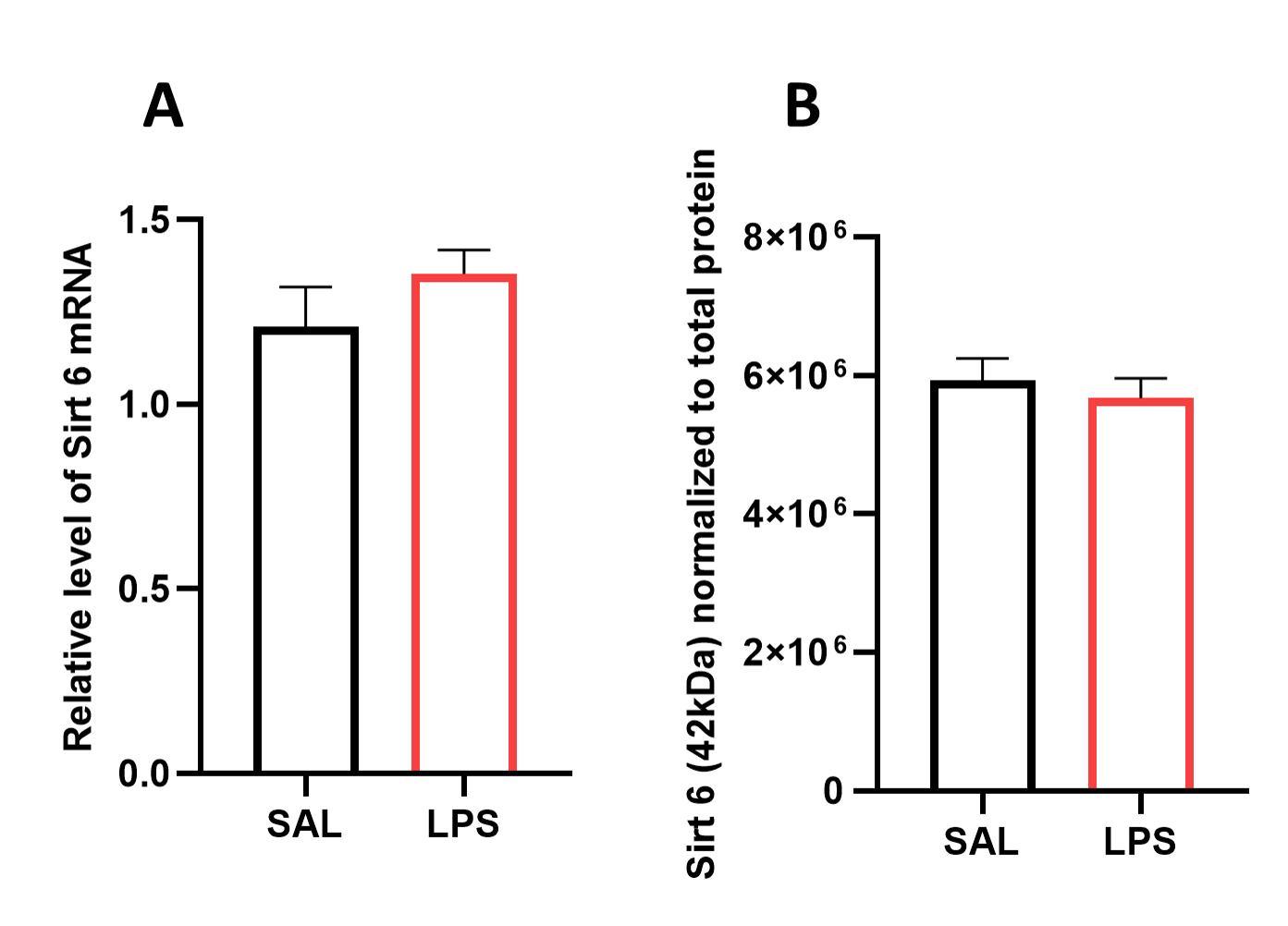

Supplement: Supplementary Figure 1 — Sirtuin 6 expression in the brain after lipopolysaccharide (LPS) treatment. Sirtuin 6 mRNA expression in the brain was assessed by (A) qPCR and protein expression by (B) Western blot 12 h after LPS. n = 10–17/group. Independent t-test was performed. [file Image_1.TIF]
